# Genome-wide association mapping of frost tolerance in barley (*Hordeum vulgare* L.)

**DOI:** 10.1186/1471-2164-14-424

**Published:** 2013-06-27

**Authors:** Andrea Visioni, Alessandro Tondelli, Enrico Francia, Alexander Pswarayi, Marcos Malosetti, Joanne Russell, William Thomas, Robbie Waugh, Nicola Pecchioni, Ignacio Romagosa, Jordi Comadran

**Affiliations:** 1 Centre UdL-IRTA, Departament de Producció Vegetal i Ciència Forestal, Universitat de Lleida, Lleida, Spain; 2 Consiglio per la Ricerca e la Sperimentazione in Agricoltura, Genomics Research Centre, Fiorenzuola d’Arda (PC), Italy; 3 Dipartimento di Scienze della Vita, Università di Modena e Reggio Emilia, Reggio Emilia, Italy; 4 Current address: Department of Agricultural, Life and Environmental Sciences, University of Alberta, Edmonton, Canada; 5 Biometris Applied Statistics, Wageningen University, Wageningen, The Netherlands; 6 The James Hutton Institute, Invergrowie, Dundee, UK

**Keywords:** GWAS, Frost tolerance, Barley

## Abstract

**Background:**

Frost tolerance is a key trait with economic and agronomic importance in barley because it is a major component of winter hardiness, and therefore limits the geographical distribution of the crop and the effective transfer of quality traits between spring and winter crop types. Three main frost tolerance QTL (*Fr-H1*, *Fr-H2* and *Fr-H3*) have been identified from bi-parental genetic mapping but it can be argued that those mapping populations only capture a portion of the genetic diversity of the species. A genetically broad dataset consisting of 184 genotypes, representative of the barley gene pool cultivated in the Mediterranean basin over an extended time period, was genotyped with 1536 SNP markers. Frost tolerance phenotype scores were collected from two trial sites, Foradada (Spain) and Fiorenzuola (Italy) and combined with the genotypic data in genome wide association analyses (GWAS) using Eigenstrat and kinship approaches to account for population structure.

**Results:**

GWAS analyses identified twelve and seven positive SNP associations at Foradada and Fiorenzuola, respectively, using Eigenstrat and six and four, respectively, using kinship. Linkage disequilibrium analyses of the significant SNP associations showed they are genetically independent. In the kinship analysis, two of the significant SNP associations were tightly linked to the *Fr-H2* and *HvBmy* loci on chromosomes 5H and 4HL, respectively. The other significant kinship associations were located in genomic regions that have not previously been associated with cold stress.

**Conclusions:**

Haplotype analysis revealed that most of the significant SNP loci are fixed in the winter or facultative types, while they are freely segregating within the un-adapted spring barley genepool. Although there is a major interest in detecting new variation to improve frost tolerance of available winter and facultative types, from a GWAS perspective, working within the un-adapted spring germplasm pool is an attractive alternative strategy which would minimize statistical issues, simplify the interpretation of the data and identify phenology independent genetic determinants of frost tolerance.

## Background

The genetic components underlying the response of plants to the photo-thermal environmental cues affecting seasonal and local adaptation are key factors that limit crops’ geographical distribution and yield potential. Therefore, as they have critical implications for agricultural productivity, such components have become an important focus of applied research. The winter hardiness of temperate cereals refers to the ability of plants to withstand the chilling and freezing temperatures that occur during the winter season (cold tolerance), and is also associated with vernalization requirement and photoperiod sensitivity, to maximise yield potential whilst minimising the risk of damage due to abiotic stresses. In economically important temperate cereals, like wheat and barley, the range of cultivated germplasm can be divided into winter, facultative and spring types, depending upon their requirement for vernalization to flower and set seed. In particular, facultative genotypes, although cold tolerant and able to survive winters, can be sown in spring as well and set seed without vernalizing [1]. The genetic control of frost tolerance is complex and is the final manifestation of several component traits. Plants become tolerant to the effects of freezing through the acclimation process or *hardening,* i.e. a relatively slow, adaptive response during late autumn, signalled by a gradual decrease in temperature, day length and light intensity leading to a series of biochemical changes enhancing the cold resistance of sensitive tissues. Frost tolerance also depends on the intrinsic capacity of the vegetative tissues to survive freeze-induced desiccation, and their efficient recovery from the stress [2].

In the Triticeae, major efforts to dissect the genetic basis of winter hardiness component traits, i.e. frost tolerance and induction of flowering by temperature and day length, have been mainly based on the use of bi-parental mapping populations of random recombinant lines in wheat and barley. The bi-parental studies led to the discovery of mostly major loci responsible for the traits. Major quantitative trait loci (QTLs) emerged as logical targets for gene cloning, and the causal candidate genes have been identified for many of them [*Vrn-1/Fr-1* and *Vrn-2*[3-5], *Ppd-1*[6], *Ppd-2*[7] and *Fr-2*[8,9]]. In the case of frost tolerance, the literature in barley is scarce with most of the original QTL studies confounded with segregation of one or more major developmental genes. The ‘Nure’ (winter) x ‘Tremois’ (spring) barley population [10] is at present the only example of biparental mapping in the Triticeae where both *Frost Resistance-H1*, *Fr-H1*, and *Frost Resistance-H2*, *Fr-H2* segregated and were located on a genetic map, approximately 30 cM apart on the long arm of chromosome 5H. A cluster of at least 13 C-repeat Binding Factor (*CBF*) genes [8] co-segregates with the barley (*Fr-H2*) and the wheat (*Fr-*A2) orthologous loci [10,11]. The *CBF* gene family has been shown to have a critical role in stress response in Arabidopsis and encodes a small family of transcription factors that have been demonstrated to regulate cold acclimation response by controlling the level of COR (cold-regulated) expression, which in turn promotes tolerance to freezing. In barley, it has been shown that variation in both expression levels and copy numbers of *CBF* genes is associated with low temperature tolerance differences amongst cultivars [12]. *Fr-H1* co-segregates with *HvBM5A*, the barley candidate gene for *Vrn-H1*, a major developmental locus governing barley vernalization requirement. Allelic variation at the wheat *Vrn-A1* locus is sufficient to trigger the regulatory cascade that down-regulates the cold acclimatisation pathway [13]. A new major low temperature tolerance QTL, designated *Fr-H3*, has recently been discovered on barley chromosome 1H in the ‘NB3437f’ (facultative) × ‘OR71’ (facultative) and the ‘NB713’ (winter) × ‘OR71’ barley populations [14] but the gene underlying *Fr-H3* has yet to be identified. Other loci with minor effects on freezing tolerance at the vegetative stage have been mapped on barley chromosomes 1HL, 4HS and 4HL in the ‘Dicktoo’ (facultative) × ‘Morex’ (spring) barley mapping population together with *Fr-H1*[15]. A locus for frost induced sterility at the reproductive stage has been mapped distally on barley chromosome 2HL in the ‘Haruna Nijo’ (facultative) × ‘Galleon’ (spring) barley mapping population [16-18]. All these populations involve genetically broad crosses between winter (or facultative) and spring growth habit parents, which therefore segregate for *Vrn-H1* and/or *Vrn-H2* (major genes governing the vernalization requirement in the cultivated barley genepool) [19] that may obscure and complicate interpretation of the results.

Although bi-parental mapping has been successful in identifying key genetic switches affecting frost tolerance in barley, it can be argued that both the limited size and the genetic origin of the mapping populations capture only a portion of the genetic diversity of the species. Furthermore, the usually large pleiotropic and epistatic effects involving major genes segregating within bi-parental crosses limit our capacity to detect other loci with smaller effects and significant interactions amongst them and the environment.

The emergence of high throughput SNP marker genotyping platforms enabled the implementation of genome-wide association studies (GWAS) in crop plants, where the interest is considerable [20,21]. Barley is a diploid autogamous crop plant where linkage disequilibrium (LD) is predicted to be extensive [22-25]. Therefore medium-resolution GWAS can potentially be used to capture significant genetic effects segregating in the cultivated gene-pool [25] and successful examples considering simply inherited traits have recently been published [26,27]. Both these studies demonstrated that there is enough accumulated recombination within the cultivated gene-pool to identify and functionally validate the candidate genes responsible for the traits. For instance, Cockram et al. (2010) used a collection of 500 elite UK barley lines genotyped with 1536 SNP markers to identify the causal polymorphism for *ANT-2*, a major switch governing anthocyanin production in barley. Ramsay et al. (2011) used a genetically broader germplasm collection consisting of 192 American / European elite cultivars genotyped with 4608 SNP markers to identify the candidate gene for *INT-C*, one of the genes controlling barley spike morphology. The candidate gene was then validated using a collection of well-characterized mutant stocks [27,28]. Comadran et al. (2012) used a genome-wide scan for divergent selection footprints in 216 spring and 207 winter two-rowed barley genotyped with the iSelect 9k SNP platform to identify the candidate gene for *EARLINESS PER SE* 2 (*EPS2*), a locus associated with spring growth habit and environmental adaptation in barley, which was validated by re-sequencing the candidate gene in a historical collection of putative early flowering mutants [29].

Three association mapping approaches for studies of frost tolerance have been published to date in the Triticeae: two in barley and one in rye, the most frost tolerant species. A genome wide association study of winter hardiness traits using 3072 SNPs to genotype North American barley germplasm identified significant associations on chromosome 5H [30]. In the *Fr-H2* region, two SNPs were significantly associated with the trait, one representing the *HvCBF9* gene, and the other located in an EST encoding a heat shock transcription factor (HSF). At the *Fr-H1* locus, a specific *HvBM5A* intron 1 amplicon showed the most significant association, whereas a third significant SNP, about 8 cM proximal to *HvBM5A*, showed the highest homology to an Arabidopsis gene predicted to code Glutamyl-tRNA amidotransferase subunit C, a gene with no obvious relationship with frost tolerance. In a previous study using a different germplasm collection, the allelic variation of four out of the thirteen 5H barley *CBF* genes was studied in a panel of 216 spring and winter accessions. Two nucleotide variants of *HvCBF14* and one nucleotide variant of *HvBM5A* (barley *Vrn-H1* gene candidate) were detected as being significantly associated with frost tolerance [31]. In rye, a study of eleven candidate genes involved in frost response (including several *ScCBFs*, *ScICE2*, and *ScVRN1*) [32] found no association of frost tolerance with *ScVRN1*, although the authors did detect significant epistatic effects involving it.

Progress in understanding the genetic basis of barley tolerance to low temperatures is often hindered by the unpredictability of the number, length and intensity of the frost episodes in field conditions and the difficulty in reproducing realistic field environments under controlled environment conditions. In a recent study, we utilized a panel of barley accessions representative of the Mediterranean basin and NW Europe to study barley adaptation to drought environments by analyzing genotypic, phenotypic and environmental data from 28 sites over two years [33]. We found strong genotype × environment interactions complicate the interpretation of the data as different associations may be detected in different environments and proposed joint analysis of all the data as a way to identify and prioritize main QTL effects that were robust over a broad range of environmental conditions [33]. In this way, we identified QTLs for yield components, heading date, harvest index and plant height, many linked to known major developmental loci. During this study, we observed extremely cold winter conditions with severe and long freezing events with minimum temperatures ranging from −5°C to −10°C at the Foradada (inland Spain) trial site in 2004/5 without snow cover. In order to obtain a more reliable evaluation of cold damage, the same gemplasm panel was assessed for winter survival in the growing season 2007/8 at Fiorenzuola d’Arda (northern Italy), where it was again subjected to cold stress. The data from these two trials represent a valuable opportunity to identify important genetic regions associated with frost tolerance in barley by GWAS across a broad range of germplasm and to advance our understanding of the character.

## Results

### Environmental conditions and natural variation for frost tolerance in the association mapping panel

**Figure 1:**
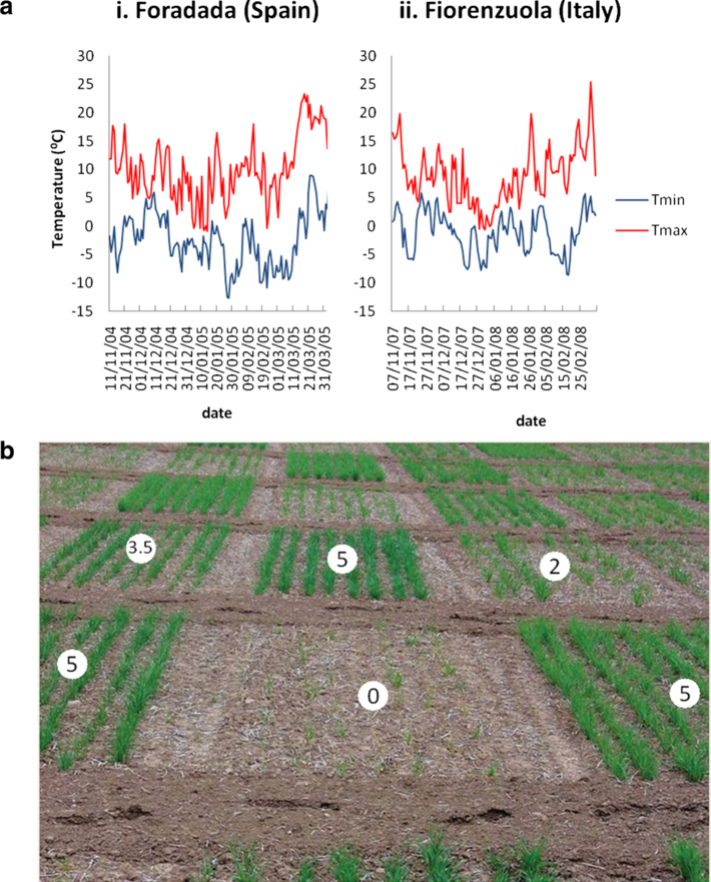
**Frost episodes in Foradada (Spain, winter 2004/05) and Fiorenzuola (Italy, winter 2007/08). a**. Daily maximum and minimum temperature from sowing to the end of March when cold tolerance was scored. The average mean minimum temperatures were −2.7°C and −1.1°C for Foradada (**i**) and Fiorenzuola (**ii**) trials respectively, while the absolute lowest temperatures were −12.7°C and −8.6°C for Foradada and Fiorenzuola trials respectively. An alternation of freezing and thaw periods was observed. **b.** Differential frost damage in winter barley plots (Foradada, Spain spring 2005). Cold injury was visually estimated on a 0–5 scale: 0. all plants killed; 1. whole plants yellowed and 50% of plant mortality; 2. whole plants yellowed and 20% of plant mortality; 3. fully yellowed basal leaves; 4. half yellowed basal leaves; 5. no damage.

Minimum and maximum temperatures recorded in Foradada trial during the 2004/5 winter show the severity of the frost episodes experienced by the panel, with long and extreme freezing periods, where minimum temperatures below 0°C were recorded for several consecutive weeks (Figure 1a.i). The 2007/8 winter in Fiorenzuola was less severe with minimum temperatures oscillating between +5°C and -5°C (Figure 1a.ii). The average visual scores recorded for ‘Nure’ (4) and ‘Tremois’ (2) in Fiorenzuola on the 0–5 scale were very similar to their multi-annual averages in the same location. Repeatability (H^2^) of cold tolerance measures in both trials was high (H^2^ = 0.84 and 0.81 for Foradada and Fiorenzuola, respectively).

As expected, analysis of variance of the trait revealed highly significant main effects of germplasm type (landraces, old cultivars and elite cultivars), geographic origin, ear type, and seasonal growth habit (Additional file 1: Table S1), but no significant interactions were detected between these classifications (data not shown). Winter lines are generally more tolerant to freezing temperatures than spring types but great fluctuation of low temperature tolerance values can be observed amongst the spring lines, consistent with little selection pressure and the inclusion of facultative types in this class. Interestingly, the most frost-tolerant lines were detected amongst Turkish facultative types (Additional file 2: Table S2, Additional file 3: Table S3), which supports a report that frost tolerance in these accessions may be either due to other loci in addition to the linked pair *Fr-H1*/*Vrn-H1* and *Fr-H2* on 5H, or alternatively, more hardy alleles at one or both loci [34].

### GWA mapping

**Figure 2:**
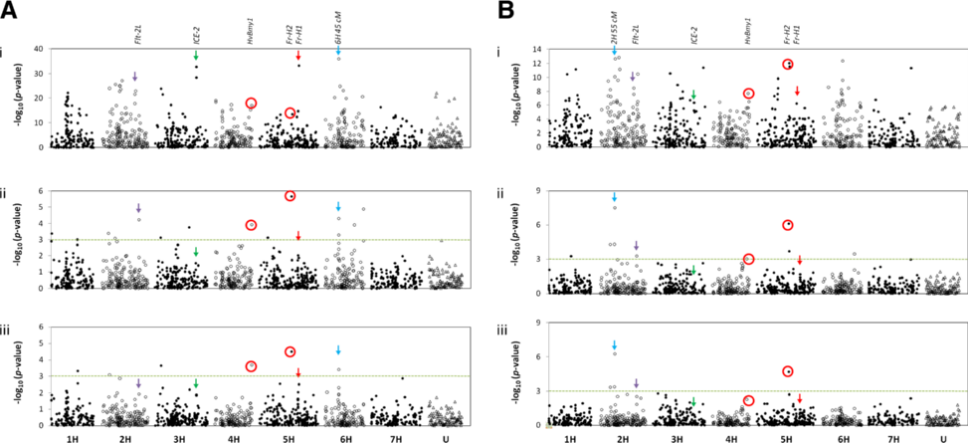
**Manhattan plots for frost tolerance in barley in the Foradada (Spain) location where the frost episode was long and severe (A) and in the Fiorenzuola (Italy) location where accessions experienced less severe cold conditions (B).** (**i**) ”Uncorrected” naive analysis. (**ii**) EIGENSTRAT and (**iii**) Kinship analysis. The -log_10_*(p-values)* from a genome-wide scan are plotted against the position on each of the 7 barley chromosomes. The horizontal line indicates the genome-wide significance threshold (−log_10_*p* ≥ 3). Three amongst the top ‘uncorrected’ hits fail to reach the significance threshold in the kinship analysis despite being genetically close to *FLT-2L-linked ’*frost sensitivity in reproductive tissues’ locus (downward purple arrow), *ICE2* regulatory gene (downward green arow) and the *Fr-H1* locus (downward red arow), known to be involved with cold tolerance. (red circle) Association hits previously reported in the literature [30]. (down ward light blue arrow) Top hit in the ‘uncorrected’ analysis also significant in the kinship analysis.

Using the Tracy-Widom statistic, we found that 18 eigenvectors were necessary to describe the population sub-structure in our germplasm panel explaining 60% of the genetic Variance within the dataset. For the Foradada dataset, 13 and 6 SNPs exceeded the significance threshold for EIGENSTRAT and kinship mixed models, respectively (Table 1a and 1b). Several of these QTLs map in the same regions as loci previously reported to be involved in cold tolerance (located on Figure 2). SNP 11_20320, located on the long arm of chromosome 5H at 108 cM, is a polymorphism in *HvCBF6* located within a physically linked cluster of at least 13 *CBF* family members (also known as *DRE binding protein 1* (*DREB1*)) that correspond to the frost resistance *Fr-H2* locus [8]. This marker is significantly associated with frost tolerance with both ‘Intro’ and ‘Tokak’ possessing the more tolerant allele. The significant association found with SNP 11_20366 on the long arm of chromosome 2H at 128 cM is in the same region as the *FLT-2L* flowering locus [35], which has also been reported to be closely linked to a QTL controlling frost induced sterility at the reproductive stage [17,18], although this association was only significant in the EIGENSTRAT analysis. At this locus, ‘Tokak’ carries the more tolerant allele and ‘Intro’ the more susceptible one. Other SNPs mapping in the region could help in detecting further recombinants and identifying putative candidate genes. SNP 11_11019, which is co-located on chromosome 4HL at 123 cM with SNP 12_30824, had the most significant association with low temperature tolerance in another GWAS of cold tolerance in barley and both SNPs are located in the same beta amylase barley unigene (*HvBmy1*) [30]. Both SNPs have been mapped ~4 cM distal to other SNPs located in the barley vernalization gene *VRN-H2*[20]. In Arabidopsis, one specific beta-amylase has been shown to have a key role for the cold-temperature dependent increase in soluble sugars and the associated protection of the photosynthetic electron transport chain and proteins in the chloroplast stroma during freezing stress [36]. A similar role could be suggested for the *HvBmy1* underlying our association hit. Both ‘Intro’ and ‘Tokak’ carry the more cold tolerant allele, which is associated with the winter allele at *Vrn-H2* (Table 1). It is important to note that ‘Tokak’, as a facultative line, does not carry the winter allele at *Vrn-*H2 [34] and in fact carries the spring allele at SNP 11_20272, which is co-located with SNPs in *Vrn-H2*. The same SNP associations on chromosomes 2H and 5H were also significant at Fiorenzuola but the significant SNP on chromosome 4H was 11_21130, approximately 2 cM proximal to *Vrn-H2*, although both ‘Tokak’ and ‘Intro’ again carried the more resistant winter allele which was in the majority. Using EIGENSTRAT, an additional 6 significant SNPs were detected at Fiorenzuola but whilst the significant SNP on 5H was also detected using kinship, no SNPs in the region of *FLT-2L* were amongst the remaining 3 significant associations (Table 1). Apart from the three above loci, all the remaining significant associations are in regions which have not previously been reported as being related to frost tolerance. The most significant hit for the Fiorenzuola trial (for both structure models) was located in the pericentromeric region of chromosome 2H at 55 cM. For the Foradada trial, two of the 13 significant EIGENSTRAT associations were co-located on chromosome 6H at 45.4 cM, with the most significant (SNP 11_10013) also being detected by kinship. ‘Intro’ and ‘Tokak’ carry the same allele at SNP 11_10013, which increases cold tolerance.

**Table 1:** **Summary of significant (−log**_**10 **_***p *≥ 3) marker-trait associations identified by genome-wide association scans where a. EIGENSTRAT analysis. b. Kinship analysis**

**Peak marker name**	**Chromosome position (cM)**		**GWA statistics**			**SNP diversity**		**Reference cultivars**	
			**-log**_**10**_**(p)**	Effect*	**(s.e.)**	**Alleles**	MAF**	**‘Tokak’**	**‘Intro’**
**a - Foradada**									
11_21067	1H	1.5	3.36	−0.20	0.06	G/A	A (0.293)	G/G	G/G
11_10216	2H	26.5	3.4	−0.21	0.06	A/C	C (0.467)	A/A	A/A
11_10498	2H	49.1	3.08	−0.36	0.11	G/A	A (0.207)	G/G	G/G
11_20366	2H	128.3	4.23	+0.31	0.08	A/G	G (0.402)	A/A	G/G
11_10565	3H	19.1	3.13	−0.27	0.08	A/G	G (0.247)	A/A	A/A
11_20168	3H	114	3.77	+0.44	0.12	G/A	A (0.103)	G/G	G/G
11_11019	4H	123.3	3.93	−0.35	0.09	A/G	G (0.125)	A/A	A/A
11_11048	5H	29.9	3.12	−0.28	0.08	T/A	A (0.147)	T/T	A/A
11_20320	5H	108.2	5.65	−0.31	0.07	A/C	C (0.310)	A/A	A/A
11_10817	6H	45.4	3.29	−0.24	0.07	C/A	A (0.473)	A/A	C/C
11_10013	6H	45.4	4.29	−0.40	0.1	A/G	G (0.212)	A/A	A/A
11_20531	6H	97.4	3.9	+0.34	0.09	G/A	A (0.136)	G/G	A/A
11_11111	6H	128.5	4.88	+0.35	0.08	A/G	G (0.332)	A/A	G/G
**a -Fiorenzuola**									
11_21126	1H	73.9	3.25	−0.20	0.06	G/C	C(0.152)	G/G	C/C
11_10919	2H	39.1	4.29	−0.21	0.05	G/A	A(0.462)	G/G	G/G
11_11522	2H	53.5	4.32	−0.27	0.07	A/G	G(0.185)	A/A	A/A
11_21388	2H	55	7.51	−0.40	0.07	A/C	C(0.141)	A/A	A/A
11_20366	2H	128.3	3.29	+0.22	0.06	A/G	G(0.402)	A/A	G/G
11_21130	4H	116.9	3.02	−0.24	0.07	C/A	A(0.158)	C/C	C/C
11_20320	5H	108.2	6.1	−0.26	0.05	A/C	C(0.310)	A/A	A/A
11_21168	5H	109.6	3.69	−0.21	0.06	G/A	A(0.440)	G/G	G/G
11_21271	6H	105.6	3.48	+0.25	0.07	C/A	A(0.321)	A/A	C/C
**b - Foradada**									
11_21192	1H	88.2	3.31	−0.27	0.08	A/T	T (0.277)	A/A	A/A
11_21187	2H	29.2	3.08	−0.33	0.1	G/A	A (0.141)	G/G	G/G
11_10565	3H	19.1	3.65	−0.32	0.09	A/G	G (0.247)	A/A	A/A
11_11019	4H	123.3	3.68	−0.38	0.1	A/G	G (0.125)	A/A	A/A
11_20320	5H	108.2	4.48	−0.30	0.07	A/C	C (0.310)	A/A	A/A
11_10013	6H	45.4	3.4	−0.39	0.11	A/G	G (0.212)	A/A	A/A
**b -Fiorenzuola**									
11_10919	2H	39.1	3.33	−0.19	0.06	G/A	A(0.462)	G/G	G/G
11_11522	2H	53.5	3.37	−0.26	0.07	A/G	G(0.185)	A/A	A/A
11_21388	2H	55	6.26	−0.40	0.08	A/C	C(0.141)	A/A	A/A
11_20320	5H	108.2	4.69	−0.25	0.06	A/C	C(0.310)	A/A	A/A

The Turkish low temperature tolerant Facultative line ‘Tokak’ and the elite winter two-rowed cultivar ‘Intro’ are used to illustrate typical cultivars genotypes with maximum cold tolerance scores in our trial conditions. * Allele effects for the minor allele. ** *MAF* Minor allele frequency.

The most significant SNP detected by GWAS of the Foradada phenotypes without population structure correction was located within 5 cM of *HvBM5a*, the barley vernalisation locus *Vrn-H1* (Figure 2), which is also considered the candidate gene for *Fr-H1,* on chromosome 5H at 137 cM [1,10,13,30]. As we would expect *Fr-H1* to be involved in frost tolerance, we can conclude that this is probably a genuine effect that is confounded with population sub-structure. As expected, the frost tolerant facultative and winter cultivars [34] ‘Tokak’ and ‘Intro’ carry the more cold tolerant allele. Two other highly significant associations were found for SNPs 11_21428 and 11_20409, located on chromosome 3H at 136.66 cM, when analysing the data without correction for sub-structure. Based on synteny and colinearity with rice sequence data, these two SNPs are located 11 and 14 genes away from *INDUCER of CBF EXPRESSION 2* (*ICE2*), a known regulatory gene of the cold tolerance pathway in Arabidopsis. ‘Tokak’ and ‘Intro’ again carried the more resistant winter allele, showing an effect of 0.30 in the Foradada kinship analysis. The more resistant allele notably was in the majority, despite the winters comprising just 25% of the lines. Interestingly, an association mapping study of frost tolerance in rye using a candidate gene approach identified a rye *ICE2* homologue SNP as the most significant hit in field trials [32]. The barley homologue of *ICE2* was mapped in the same position in a previous study [37]. Although still significant, the association of SNPs 11_21428 and 11_20409 with the Fiorenzuola phenotypes was not so strong using an uncorrected GWAS. A more highly significant association was detected with SNP 11_11410, which is located distal on chromosome 3H at 167.77 cM (Figure 2). Clearly, frost tolerance is subject to Genotype x Environment interactions, which might be related to the severity of the stress. Such effects will be reflected in QTL x Environment interactions leading to SNPs being significant at one site but not the other.

### QTL frequencies and Linkage Disequilibrium (LD)

**Figure 3:**
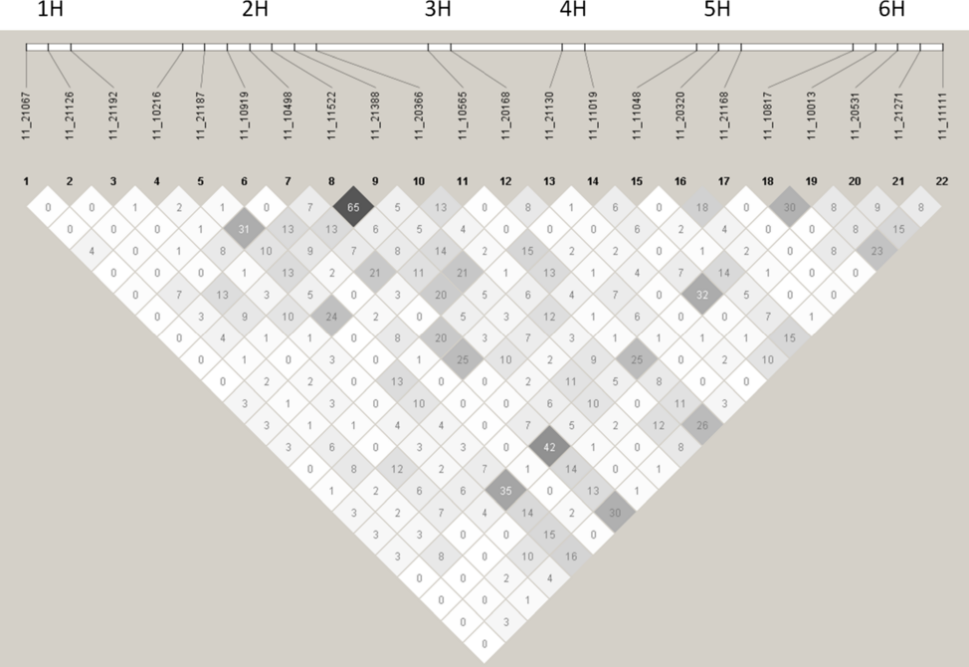
**Linkage Disequilibrium (LD) amongst significant SNP markers.** HAPLOVIEW v.4.2 (http://www.broadinstitute.org/haploview/haploview) pairwise LD values (r^2^*100) for 22 SNPs based on genotypes of 184 individuals were used to test whether all the SNPs significantly associated with frost tolerance were in strong LD with each other.

Classic LD parameters (D’ and r^2^) as implemented by HAPLOVIEW v.4.2 (http://www.broadinstitute.org/haploview/haploview) were used to test whether the significant SNPs were in strong LD with each other. The presence of very strong LD would raise concerns about a high rate of false positives present in our results whilst complete absence of LD between the positive SNPs would provide evidence of complete independence between QTLs. We only observed significant LD between SNPs 11_11522 and 11_21388 (r^2^= 0.65), both located on chromosome 2H at 53.5 and 55 cM, respectively, suggesting their effects are not independent. For the rest of the SNPs we did not observe signs of strong inter-QTL LD resulting from population substructure and admixture within our panel even for closely linked significant SNPs detected at Foradada such as 11_10817 and 11_10013 both mapping on chromosome 6H at 45 cM (r^2^= 0.30). With an average r^2^ value of 0.06 for all 231 possible combinations between the 22 significantly associated SNPs, most observed r^2^ values were <0.30. Only four, which were amongst the six possible combinations between SNPs 11_21187, 11_10498, 11_11019, and 11_10013, were greater than 0.3 and none exceeded 0.5 (Figure 3). Such r^2^ values are not strong enough to suggest non-independence between QTL regions but they might add some evidence of co-selection, a reasonable situation when measuring traits of high agronomic/economic importance subjected to a long history of breeding and selection. For example, despite a contrasting breeding origin, ‘Intro’ and ‘Tokak’ possess the same alleles at all the significant SNPs associated with frost tolerance in the kinship model (Table 1).

**Figure 4:**
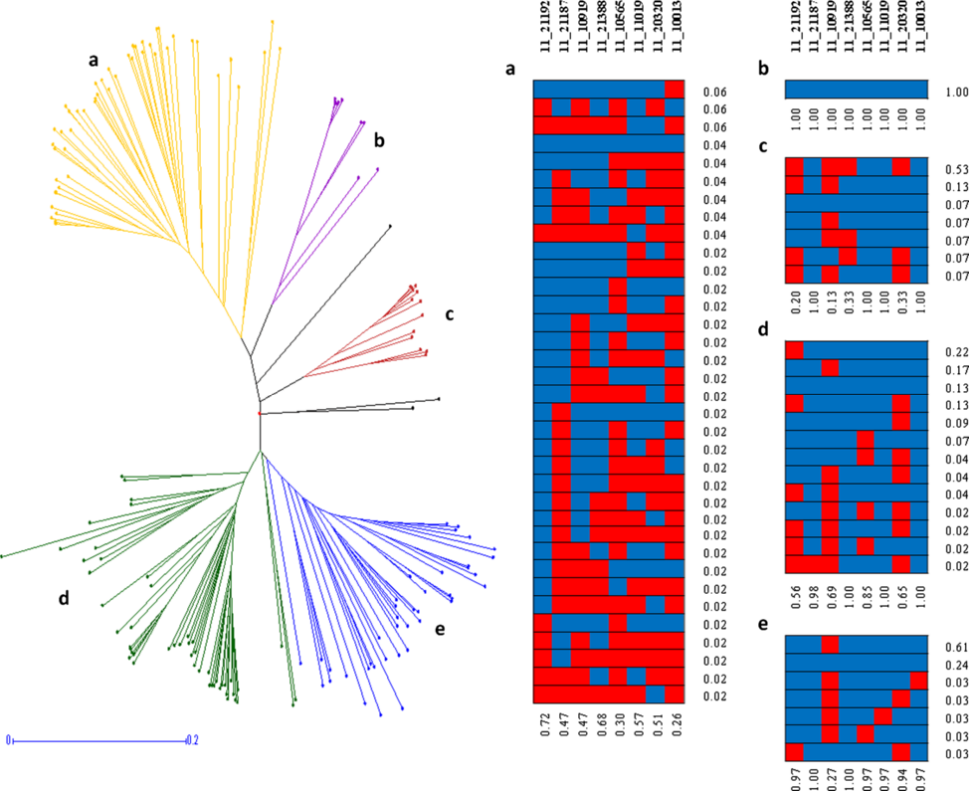
**QTL haplotype analysis across germplasm clusters.** (1) Left figure: Neighbour joining tree of the selected 184 barley cultivars constructed from simple matching distance of 1,307 SNP markers. Lines are coloured according to population structure clusters described in Comadran et al. 2009: **a.** Northern European springs; **b.** Turkish; **c.** Syrian and Jordan; **d.** South-West Mediterranean accessions; **e.** Northern European winters. (2) Right figure: QTL and QTL haplotype frequencies within population structure clusters for the eight significant SNPs detected with the kinship analysis in the Foradada and / or the Fiorenzuola field trials (Table 1). Reported allele frequencies correspond to the SNP allele increasing winter survival (blue). The alternative allele is shown in red.

In order to understand which genotypes possess favourable frost tolerance alleles, we examined QTL diversity and distribution in the different genetic clusters of our GWA panel. We evaluated the genetic relationships among the accessions by generating a neighbour-joining population tree based on simple matching of allelic distances as implemented in DARwin v.5.0 [38], which produced clearly separated branches corresponding to each of our germplasm origin groupings: (a) 51 Northern European springs, (b) 18 Turkish, (c) 15 Syrian and Jordan, (d) 59 South-West Mediterranean accessions and (e) 42 Northern European winters (Figure 4). This analysis provided the same groupings as the Bayesian cluster analysis implemented in the program STRUCTURE [39] and Principal Coordinates results described in previous publications [23,33]. Subsequent QTL haplotype analysis for the eight significant SNPs detected with the kinship analysis in the Foradada and/or the Fiorenzuola field trials highlighted a large number of different allelic combinations within the ‘Northern European springs’ germplasm, with one specific combination of the QTL alleles fixed or nearly fixed in both winter clusters (‘Northern European winters’ and ‘South-West Mediterranean’ lines). A high degree of allelic fixation was also observed within the cold tolerant ‘Turkish’ and ‘Syrian and Jordan’ clusters (Figure 4).

## Discussion

Genome wide association analysis has arisen as a powerful tool to dissect quantitative traits as it potentially provides higher resolution QTL mapping than bi-parental studies and is therefore of interest to both the academic and commercial sectors [21,40]. A recent GWAS study using 148 advanced breeding barley lines identified QTLs for frost tolerance in the region of *Fr-H1* and *Fr-H2* but the combined effect of both loci explained just 25% of the phenotypic variation, suggesting undetected genetic variation elsewhere [30]. The study [28] utilized advanced breeding material where linkage disequilibrium (LD) was extensive; r^2^ LD values higher than 0.6 persisted for 5 cM and 15 cM around *Fr-H2* and *Fr-H1*, respectively. Strong LD values across long genetic distances such as those found in the region of *Fr-H2* and *Fr-H1* usually arise from inbreeding and recent selection (usually within breeding programs) and can be an indication of strong population stratification. Both aspects affect random segregation of alleles within the genome and constitute real handicaps for association mapping studies.

Studies sampling advanced breeding material could be severely limited by the effects of inbreeding and/or selection. Our approach of using landraces and old cultivars from distinct geographical origins samples a longer history of recombination events, which will dilute the effects of LD related to inbreeding. However, by sampling spring, facultative and winter genotypes, we inevitably introduced sources of population structure and the need to account for them in our analyses. We accounted for population structure in the analysis by using a mixed linear model with either the Eigenstrat relationship model with PCA scores as a random terms or a kinship matrix to correct for population substructure. For comparison, we also conducted GWAS without any correction for population substructure, as we believed that some critical genes may well be confounded with the population sub-structure existing in our germplasm panel. Current barley genome map coverage and knowledge about the role of *Fr-H1* and *Inducer of CBF expression 2, ICE2,* in cold tolerance [32] suggests that the strong associations in the “uncorrected” approach tightly linked to both loci represent true genetic effects that need to be included in improvement of frost tolerance rather than false positives introduced by the absence of population structure correction in the analysis (Figure 2). Similarly, the *FLT-2L* related hits (SNP 11_20366) - with extensive literature on the involvement of this genomic region in frost sensitivity in reproductive tissues [16-18] - in both Foradada and Fiorenzuola EIGENSTRAT analyses do not reach the statistical threshold using the kinship approach, which is the most conservative method of analysis of the three that we adopted. Correction for population structure in GWAS performed in highly stratified populations can therefore result in important associations being undetected. Whilst it is necessary to avoid an inflated rate of false positives arising from the genetic stratification of the germplasm, we have found that true associations might not be detected due to a complete confounding with the population structure of the germplasm. In this example, 2 of the 3 top hits (tightly linked to major, known genetic determinants of cold tolerance) identified in the uncorrected approach fail to reach the significance threshold in both EIGENSTRAT and kinship analyses.

The most conservative of our GWAS analyses (kinship) detected six and four significantly associated SNPs for frost tolerance from the Foradada and Fiorenzuola trials respectively. SNP 11_20320, linked to *Fr-H2* (*HvCBF* cluster), was detected in both trials (Table 1) and was the most significant association detected under the severe cold conditions experienced in Foradada in 2004/5. Although *Fr-H2* was also significant in the milder winter conditions experienced in Fiorenzuola in 2007/8, it is interesting that the most significant association for this environment was found for SNP 11_21388, located in the pericentromeric region of chromosome 2H (55 cM), that has not previously been reported. This region of the genome is, however linked to varying degrees to the more distal *Ppd-H1* (located on chromosome 2HS at 26.6 cM) and the earliness per se locus *EPS-H2* locus (located at 63 cM on chromosome 2H) [29]. The history of selection in regions such as this one with major developmental genes often leaves complicated linkage disequilibrium patterns which add confusion to the interpretation of results. ‘Intro’ and ‘Tokak’ carry the more frost tolerant alleles at the 11_21388 locus and ‘Intro’ also carries the earlier flowering allele at *EPS-H2* so, one would think that earlier flowering would make a plant more susceptible to frost damage. Although study of LD between SNPs representing *Ppd-H1* and *EPS-H2* and 11_21388 shows that they are in equilibrium and that we may have identified a genuine effect and not something that is confounded with maturity differences at *Ppd-H1* and *EPS-H2,* further research is necessary to properly characterise this region. The other novel regions detected are also interesting findings that are worthy of future research.

Exploration of the allelic frequencies of the significantly associated SNPs detected by kinship analysis revealed that the more frost tolerant allele at most of the QTL is genetically fixed or nearly fixed within the winter barley germplasm (Figure 4e). The more frost tolerant alleles are also nearly fixed or fixed within the Syrian and Jordan landraces (Figure 4c) and the Turkish facultative lines (Figure 4b) respectively, both known to be winter hardy. In contrast, most QTLs are freely segregating within the spring germplasm (Figure 4a) with a noticeably greater range of haplotypes and none of the 35 observed (from 256 possible combinations) dominating. The South-West Mediterranean lines represent an intermediate situation with four more dominant (frequency >0.1) haplotypes but definitely much more variation than the other autumn sown groups (Figure 4d). Two noteworthy conclusions can therefore be drawn. Firstly, the majority of spring × winter crosses will sample variation in frost tolerance that is mostly dependent upon the genetic make-up of the spring parent. Secondly, working within diverse un-adapted spring germplasm pools is a useful alternative strategy as it would avoid complications associated with the pleiotropic effects of major developmental genes segregating in spring x winter crosses, and would minimize the statistical issues relative to the inherent population structure of the germplasm. Clearly, whilst true winter genotypes represent just over 25% of our germplasm panel, the frost tolerant alleles are generally the most frequent (Table 1). It is also highly possible that the cold adapted winter gene pool contains additional alleles that were not detected by our analyses. Although we might expect such effects to be minor, we have sampled a limited number of environments yet highlighted the potential importance of QTL × Environment interactions. Future work should be directed towards suitably augmenting the genetic diversity of the panel and increasing the environmental diversity to which it is exposed in order to obtain as complete as possible survey of frost tolerance genetics. The information we have generated is also valuable to choose parental lines to use in crosses (in the form of bi-parental or MAGIC populations) to complement the GWAS data and analyse some of the novel associations in more detail. However, as the genetic determinants underlying major loci *Fr-H1* and *Fr-H2* have already been identified, population numbers will need to be increased in order to capture effects that can be masked by the pleiotropic effects of such major loci*.* Moreover, larger populations will be useful to detect epistatic effects that have naturally been accumulating in cultivated germplasm, but will probably be undetected in bi-parental populations due to the random effects of segregation without selection.

An attractive alternative which does not involve the development of new plant material to be tested involves the ultra-saturation of the genome by emerging new generation sequencing technologies such as genotyping by sequencing (GbS) [41,42]. These technologies promise a deeper coverage of polymorphic sequence information and therefore increase the likelihood of identifying polymorphisms more closely linked to a character, if not the causal polymorphism itself, thus providing a manageable shortlist of gene candidates to be functionally tested. At the same time, techniques like GbS provide an unbiased assessment of the diversity at every single gene sampled, which will result in a fast implementation of the results arising from those data in the form of specific haplotype and allele information tightly linked to the regions of interest, facilitating the direct identification of the causal polymorphism. These could be efficiently used as new molecular tools in marker assisted selection (MAS) programs, aiming to accumulate the frost tolerant alleles in acceptable genetic backgrounds to be used in breeding programmes and potentially increase the overall frost tolerance of barley.

## Conclusions

Progress in understanding the genetic basis of barley tolerance to low temperatures is often hindered by the unpredictability of the number, length and intensity of the frost episodes in field conditions and the difficulty in reproducing realistic field environments in controlled experiments. Frost tolerance GWAS in field conditions from two trial sites experiencing extreme cold conditions provided invaluable data for the genetic dissection of the trait in barley. Two of the significant associations were tightly linked to the *Fr-H2* and *HvBmy* loci on chromosomes 5H and 4HL, respectively. The other significant associations were located in genomic regions that have not previously been associated with cold stress. However, some critical genes may well be confounded with the population sub-structure existing in our germplasm panel GWAS conducted without any correction for population substructure identified significant associations tightly linked to *Fr-H1, FLT-2L* and *Inducer of CBF expression 2, ICE2* loci on chromosomes 5H, 2HL and 3HL, respectively. Thus, providing evidence that correcting for population structure in highly stratified populations can result in important associations being undetected. Linkage disequilibrium and haplotype analyses of the kinship significant SNP associations showed they are genetically independent and that most of the significant SNP loci are fixed in the winter or facultative types, while they are freely segregating within the un-adapted spring barley genepool. Although there is a major interest in detecting new variation to improve frost tolerance of available winter and facultative types, from a GWAS perspective, working within the un-adapted spring germplasm pool is an attractive alternative strategy which would minimize statistical issues, simplify the interpretation of the data and identify phenology independent genetic determinants of frost tolerance.”

## Methods

### Plant material and phenotyping

The initial germplasm panel consisted of 192 genotyped barley accessions representing a survey of the breeding history of the Mediterranean basin, as well as of NW Europe and was described in full by Comadran et al. (2009).

The whole set of accessions was sown in November 2004 in Foradada (Spain, 41°39’N, 01°29’E) following an augmented cyclical design with an incomplete block size of 60. Each incomplete block was planted in 5 rows of 12 columns and included 4 checks, each replicated three times with one located in each column in a diagonal fashion at fixed intervals. We used four incomplete blocks to sow one of full replicate of all 192 entries and used a fifth incomplete block with a random selection of 48 entries to provide partial replication. The four checks (a local landrace, a local old variety, a local modern variety and the improved variety ‘Rihane’) were used to detect and correct for any spatial variation across rows and column and the partial replication provided an estimate of the trial error. Plots were 6 m^2^ and were grown according to local management practice in terms of sowing rate, weed and disease control, and fertilizer inputs. Minimum and maximum temperatures were recorded daily during the growing season (Figure 1a). Winter survival was evaluated at the end of March 2005 by visual estimation on a 0–5 scale (5 = complete survival), using half-scores when needed, as described in Figure 1b [34]. The experiment was repeated in the growing season 2007/08 at Fiorenzuola d’Arda (Italy, 44°55’N, 9°53’E) but differed in that each entry was sown in plots of two 1m long rows in a two-replicate randomised complete block design and the same observations were recorded. The entries included the cultivars ‘Nure’ (winter) and ‘Tremois’ (spring), which have been extensively trialled at the research station of Fiorenzuola d’Arda and represent standards of frost tolerance and susceptibility respectively, that can therefore be used to judge the severity of stress.

### Genotyping

As four genotypes (‘Hanna’, ‘Pioneer’, ‘Regina’ and ‘Tipper’) were clearly phenotypically different from what they were expected to be and two (‘Zephyr’ and a Jordanian landrace) were duplicated in the panel, we extracted 186 DNA samples from leaf tissue of two-week-old single plants using the DNeasy Plant DNA miniprep kit (Qiagen, Hilden, Germany), and genotyped them with Barley Oligo Pooled Array 1 (BOPA1, consisting of 1536 SNPs) using the Illumina GoldenGate platform [20]. We adopted the genetic map published with the BOPA1 platform as the genetic framework for the association analyses [20]. Furthermore, genotypes and SNP markers with more than 10% of missing data and minimum allele frequency (MAF) <10% were removed from the dataset and omitted from further analyses, leaving a data set for further analysis consisting of 184 accessions (‘Alexis’ and ‘Tichedrett’ were removed) with genotypic data for 1,307 SNPs. The QMVREPLACE procedure, implemented in Genstat v.14 (VSN International), which replaces missing marker scores with one scores from the most similar genotype(s), was used to impute missing genotypic data, using the default values. The Turkish two-rowed facultative line ‘Tokak’ and the elite two-rowed winter line ‘Intro’ are used in the results and discussion sections as reference cultivar genotypes with maximum cold tolerance scores in our trial conditions.

### Statistical analysis and GWA mapping

DARwin v.5.0 [38] (http://darwin.cirad.fr/) was used to construct a Neighbour Joining Tree from the genotypes of the 184 barley accessions using simple matching of the SNPs. Linkage Disequilibrium and haplotype analyses of the positive SNPs in relation to germplasm clusters were performed with HAPLOVIEW v.4.2 (http://www.broadinstitute.org/haploview/haploview). Best linear unbiased predictions (BLUPs) of the cold resistance for each accession were calculated using the Restricted Maximum Likelihood (REML) directive in Genstat v.14 (VSN International). In the model, checks were fitted as a fixed effect, and columns, rows and test entries were fitted as random effects. From the variance components obtained by REML, repeatability (H^2^) was estimated as H^2^ = [σ^2^_g_ / (σ^2^_g_ + σ^2^_e_)], where σ^2^_e_, is the residual variance component and σ^2^_g_ is the genotypic variance component. The BLUPs for each accession were classified according to their geographic origin, growth habit and ear morphology (Additional file 4 Table S4), the main drivers of genetic divergence of barley germplasm, and tested for significant differences by Analysis of Variance using Genstat v.14 (VSN International). Genome Wide Association Scans (GWAS) were carried out by using a mixed linear model with terms to account for genetic relatedness due to historical population substructure and/or kinship [43]. We therefore used either the Eigenstrat relationship model with PCA scores included as a random matrix that approximates kinship [44] or a kinship matrix [43], again as a random term, to correct for population substructure in the association mapping routines implemented in Genstat v.14 (VSN International). TASSEL v. 3.0 [45] was used to estimate the kinship matrix (K) from a stratified subset of 631 random markers spaced approximately 2cM apart and with unique map positions so that we did not over-estimate sub-population divergence. For comparison, we also conducted GWAS without any correction for population substructure. In all scans, a threshold of (−log_10_*p* ≥ 3) was set for identifying significant SNP associations. Significant SNPs mapping within 5 cM of each other were considered as being linked to the same QTL and the highest was chosen as representing the QTL.

## Abbreviations

SNP: Single nucleotide polymorphism; QTL: Quantitative trait loci; CM: Centimorgan; CBF: C-repeat binding factor; COR: Cold-regulated; GWAS: Genome-wide association studies; HSF: Heat shock transcription factor; H2: Repeatability; ICE: Inducer of CBF expression; LD: Linkage disequilibrium; GbS: Genotyping by sequencing; MAS: Marker assisted selection; BLUPS: Best linear unbiased predictors; PCA: Principal components analysis; MAF: Minimum allele frequency.

## Competing interests

The authors declare they have no competing interests.

## Authors’ contributions

JC, IR, NP, RW, WT and JR conceived and coordinated the study. MM, AT, AV, and JC performed statistical and bioinformatic analyses. AT, EF and AP collected phenotypic data. All authors reviewed and contributed to draft the manuscript. All authors read and approved the final manuscript.

## Supplementary Material

Additional file 1 **Table S1.** ANOVA for frost tolerance in relation to the main drivers of germplasm genetic divergence in barley: region of origin of the germplasm, growth habit (spring/ winter), ear morphology (two-rowed / six-rowed types) and germplasm type (landraces / old cultivars / modern cultivars). Click here for file

Additional file 2 **Table S2.** Summary statistics for Foradada (Spain). Genotypic means and standard error for frost tolerance for the fixed terms in the model. Number of individuals sampled in each class in brackets. Click here for file

Additional file 3 **Table S3.** Summary statistics for Fiorenzuola (Italy). Genotypic means and standard error for frost tolerance for the fixed terms in the model. Number of individuals sampled in each class in brackets. Click here for file

Additional file 4 **Table S4.** Germplasm list of the 184 barley lines assembled into the GWAS population providing country and region of origin, growth habit, spike morphology and frost tolerance genotypic means used in this paper. Click here for file
