# Supplementary material for: Genome-wide association mapping of frost tolerance in barley (Hordeum vulgare L.)
Source: BMC Genomics. 2013 Jun 27;14:424. doi: 10.1186/1471-2164-14-424 (PMC3701572; doi:10.1186/1471-2164-14-424)
Supplement: Additional file 1 — Table S1. ANOVA for frost tolerance in relation to the main drivers of germplasm genetic divergence in barley: region of origin of the germplasm, growth habit (spring/ winter), ear morphology (two-rowed / six-rowed types) and germplasm type (landraces / old cultivars / modern cultivars). [file 1471-2164-14-424-S1.docx]

| **Fixed term** | **n.d.f.** | **Foradada (Spain)** | | **Fiorenzuola (Italy)** | |
| --- | --- | --- | --- | --- | --- |
|  |  | **s.s.** | **F pr.** | **s.s.** | **F pr.** |
| Germplasm type | 2 | 17.43 | <0.001 | 0.64 | 0.352 |
| Growth habit | 1 | 26.98 | <0.001 | 9.95 | <0.001 |
| Region of origin | 4 | 48.40 | <0.001 | 20.21 | <0.001 |
| Ear type | 1 | 7.91 | <0.001 | 3.84 | <0.001 |

**Supplementary Table 1**
