# Supplementary material for: Genome-wide association mapping of frost tolerance in barley (Hordeum vulgare L.)
Source: BMC Genomics. 2013 Jun 27;14:424. doi: 10.1186/1471-2164-14-424 (PMC3701572; doi:10.1186/1471-2164-14-424)
Supplement: Additional file 2 — Table S2. Summary statistics for Foradada (Spain). Genotypic means and standard error for frost tolerance for the fixed terms in the model. Number of individuals sampled in each class in brackets. [file 1471-2164-14-424-S2.docx]

| ***Germplasm origin*** | | ***Growth Habit*** | | ***Germplasm type*** | | **Ear type** | |
| --- | --- | --- | --- | --- | --- | --- | --- |
| ***Class*** | ***Means*** | ***Class*** | ***Means*** | ***Class*** | ***Means*** | ***Class*** | ***Means*** |
| *East Med.* (21) | *4.24* | *Winter* (46) | *4.69* | *Landrace* (80) | *4.18* | *6 Rows* (84) | *4.09* |
| *North Med.* (68) | *3.67* | *Spring* (138) | *3.62* | *Old cv.* (40) | *3.85* | *2 Rows* (100) | *3.73* |
| *South Med.* (33) | *3.99* |  |  | *Modern cv.* (64) | *3.55* |  |  |
| *Other* (38) | *3.25* |  |  |  |  |  |  |
| *Turkey* (24) | *5.00* |  |  |  |  |  |  |
| *St. Error* | *0.177* |  | *0.153* |  | *0.138* |  | *0.142* |

**Supplementary Table 2**
