# Supplementary material for: Genome-wide association mapping of frost tolerance in barley (Hordeum vulgare L.)
Source: BMC Genomics. 2013 Jun 27;14:424. doi: 10.1186/1471-2164-14-424 (PMC3701572; doi:10.1186/1471-2164-14-424)
Supplement: Additional file 3 — Table S3. Summary statistics for Fiorenzuola (Italy). Genotypic means and standard error for frost tolerance for the fixed terms in the model. Number of individuals sampled in each class in brackets. [file 1471-2164-14-424-S3.docx]

| ***Region of origin*** | | ***Growth Habit*** | | ***Germplasm type*** | | **Ear type** | |
| --- | --- | --- | --- | --- | --- | --- | --- |
| ***Class*** | ***Means*** | ***Class*** | ***Means*** | ***Class*** | ***Means*** | ***Class*** | ***Means*** |
| *East Med.* (21) | *3.36* | *Winter* (46) | *3.93* | *Landrace* (80) | *3.49* | *6 Rows* (84) | *3.59* |
| *North Med.* (68) | *3.32* | *Spring* (138) | *3.27* | *Old cv.* (40) | 3.45 | *2 Rows* (100) | *3.32* |
| *South Med.* (33) | *3.14* |  |  | *Modern cv.* (64) | *3.37* |  |  |
| *Other* (38) | *3.36* |  |  |  |  |  |  |
| *Turkey* (24) | *4.36* |  |  |  |  |  |  |
| *St. Error* | *0.132* |  | *0.115* |  | *0.104* |  | *0.106* |

**Supplementary Table 3**
